# Partial results of the construction of cDNA library of *Megalopyge albicolis*

**DOI:** 10.1186/1753-6561-8-S4-P154

**Published:** 2014-10-01

**Authors:** Taisy Renata Mazur, Eneas Carvalho, Dalton Nogueira Silva Giovanni, Darci Moraes Barros-Battesti, Inácio de Loiola Meirelles Junqueira-de-Azevedo, Ursula Castro Oliveira, Ronaldo Zucatelli Mendonça

**Affiliations:** 1 Instituto Butantan, São Paulo, SP, Brazil

## Introduction

For more than 350 million years, insects live and survive in almost every ecosystem on the planet, what have resulted in the development of protection and defense mechanisms against adverse situation. This feature has stimulated research into new agents with pharmacological and biotechnology potential in the class of arthropods.

Recently, we have identified and isolated proteins of pharmacological and biotechnological interest in the hemolymph of caterpillars from family Saturnidae (*Lonomia obliqua*). Two proteins have been further characterizes: one with antiapoptotic activity and other with an antiviral action.

## Objectives

The main objective of this project is to build, characterize and compare the transcripts generated by the construction of a cDNA library of the integument of caterpillars *Megalopyge albicolis *(Megalopygidae family).

## Methods

The mRNA was isolated using the Dynabeads mRNA Direct kit (Invitrogen) and quantified with the RiboGreen RNA Reagent (Invitrogen). Subsequently, a cDNA library was produced and sequenced with a 454 GS-Junior machine (Roche). These procedures were repeated for two different tissue samples, originated from two different animals.

## Results and discussion

As a result, we obtained 38,2456 reads for the first tissue cDNA sequencing and 138,177 for the other one, with an average size of 240,66 and 439,10 bases, respectively. After the elimination of small and low quality reads, the poly A tails were trimmed off, resulting in 165,187 reads with an average size of 398,87 bases. These sequences were grouped according to their similarity using the CLC-Genomics program, resulting in 2,519 Contigs (using 118,777 reads), while the remaining 46,410 reads were classified as singlets or singletons. The more abundant transcripts are mainly related to proteins involved in catalysis, transport and binding. We also detected a high expression of the gene encoding for the protein arylphorin, with is involved in storage and in developmental and metamorphosis processes. Other frequent transcripts are also related to storage (vitellogenin) and to reactive oxygen species detoxification (catalase). These proteins need be further studied to better characterize their biological role, as well as to determinate the biotechnological potential of molecules produced by *M. albicolis*.

